# Genome Sequence of *Saccharomyces cerevisiae* Double-Stranded RNA Virus L-A-28

**DOI:** 10.1128/genomeA.00549-16

**Published:** 2016-06-16

**Authors:** Aleksandras Konovalovas, Elena Serviené, Saulius Serva

**Affiliations:** a Department of Biochemistry and Molecular Biology, Vilnius University, Vilnius, Lithuania; b Laboratory of Genetics, Nature Research Centre, Vilnius, Lithuania; c Department of Chemistry and Bioengineering, Vilnius Gediminas Technical University, Vilnius, Lithuania

## Abstract

We cloned and sequenced the complete genome of the L-A-28 virus from the *Saccharomyces cerevisiae* K28 killer strain. This sequence completes the set of currently identified L-A helper viruses required for expression of double-stranded RNA-originated killer phenotypes in baking yeast.

## GENOME ANNOUNCEMENT

Killer phenotypes among *Saccharomyces cerevisiae* were discovered and linked to cytoplasm-persisting double-stranded RNAs (dsRNAs) more than four decades ago (1). Exhaustive efforts to analyze genetic determinants behind this phenomenon led to the discovery of the interaction between two viruses belonging to the *Totiviridae* family—toxin-coding M and helper L-A—which enable the killing property. Recently, the virus Klus has been added to the group of well-described viruses responsible for the K1, K2, and K28 killer phenotypes (2). Despite the established general virus propagation scheme, evolutionary connections between the L-A and M viruses remain unresolved. In particular, cross-maintenance of dsRNAs in different killer type strains is largely unexplored, even though data on the persistence of certain combinations have begun to emerge (3).

Of the four established *S. cerevisiae* killer types, K28 clearly stands out for its unique mode of entering the target cell by endocytosis and induction of apoptosis after traveling to the nucleus (4–6). Given the importance of L-A viruses in the expression of killer phenotypes, the lack of an L-A-28 sequence precluded a full understanding of the links among yeast killer strains. Here, we report the cloning and sequencing of the complete genome of L-A-28 virus from the *S. cerevisiae* K28 killer strain (7).

Purified genomic dsRNA of the L-A-28 virus was used as a substrate for primer ligation, subsequent reverse transcription, and cDNA amplification, as described previously (8). In total, the genome of the L-A-28 virus was found to possess 4,584 nucleotides (nt). In line with other L-A family viruses, two partially overlapping open reading frames (ORFs) spanning the major part of the genome (4,517 nt) have been identified. Tentative ORF coding for the Gag-pol protein was compared with corresponding fragments of dsRNA sequences, namely, GenBank entry J04692 for the L-A-1 virus, KC677754 for L-A-2, and JN819511 for L-A-lus. At the nucleotide level, all entries display 73 to 77% identities, L-A-lus and L-A-2 being the closest relatives. Of special interest is the common frameshift area, where 100 nt in a 115-nt region match all viruses, and L-A-1 possesses 5 unique differences from the other 15 positions. Homologies of protein sequences are even higher: coat proteins (Gag) are 88 to 94% homologous and RNA polymerases (Gag-pol) are 87 to 92% homologous. In both instances, proteins originating from L-A-lus and L-A-2 are the most closely related. Therefore, the sequence of L-A-28 and those of corresponding proteins put this virus into separate phylogeny branches, L-A-1 representing one and L-A-lus with L-A-2- the other. Given the outstanding mode of action for the K28 toxin, the sequence of L-A-28 is surprisingly similar to that of other viruses of the same family; this finding provides an important evolutionary tie between L-A viruses in killer strains of *S. cerevisiae*, suggesting a common ancestor for all viruses in the Totivirus genera. The complete genome sequence of the L-A-28 virus thus completes the set for *Totiviridae* viruses involved in the expression and maintenance of all four known types of M dsRNA viruses behind the yeast killer phenotypes that have been discovered in baking yeast so far.

### Nucleotide sequence accession number.

The complete genome of *Saccharomyces cerevisiae* dsRNA virus L-A-28 has been deposited in GenBank under the accession number KU845301.
